# Supplementary figures and images for: The pesticides carbofuran and picloram alter the diversity and abundance of soil microbial communities
Source: PLoS One. 2024 Nov 26;19(11):e0314492. doi: 10.1371/journal.pone.0314492 (PMC11594414; doi:10.1371/journal.pone.0314492)

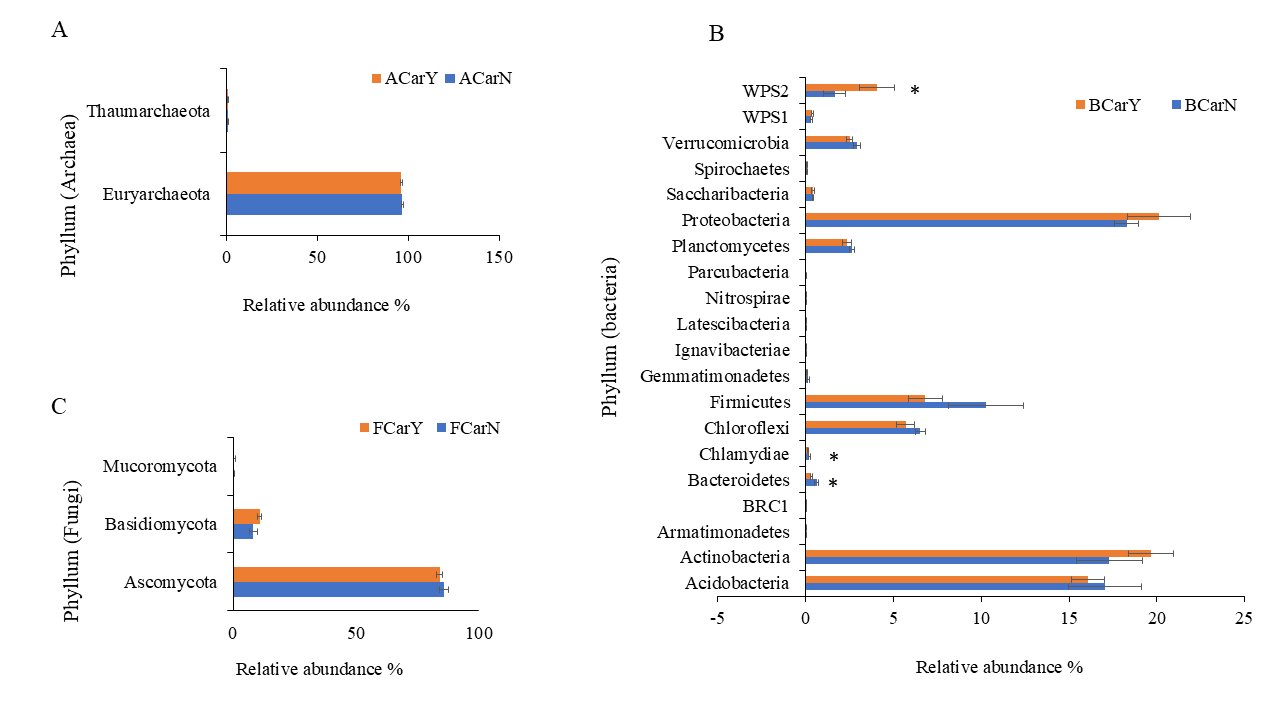

Supplement: S1 Fig — (A) Archaea; (B) Bacteria; (C) Fungi. *FDR adjusted q-value <0.05 (Pesticide—presence (CarY) vs. absence (CarN); Mann-Whitney U test). (TIF) [file pone.0314492.s001.tif]

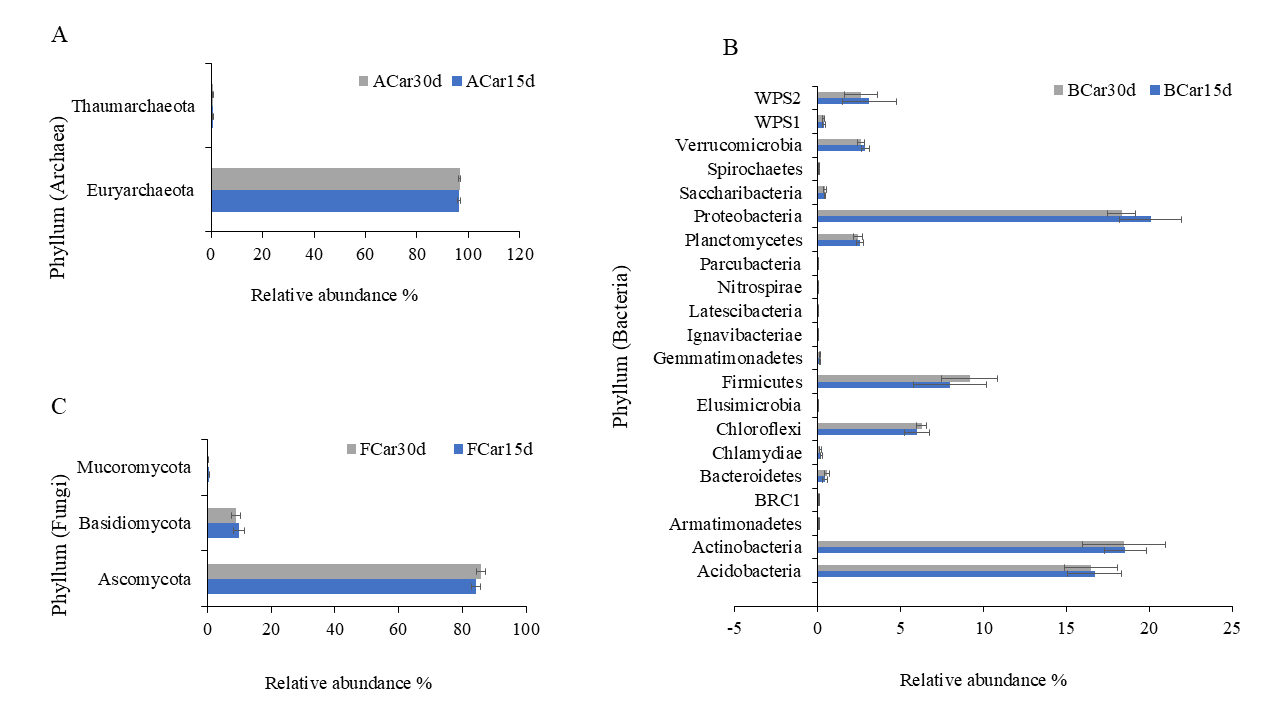

Supplement: S2 Fig — (A) Archaea; (B) Bacteria; (C) Fungi. * FDR adjusted q-value <0.05 (Time—15 days (Car15d) vs. 30 days (Car30d); Mann-Whitney U test). (TIF) [file pone.0314492.s002.tif]

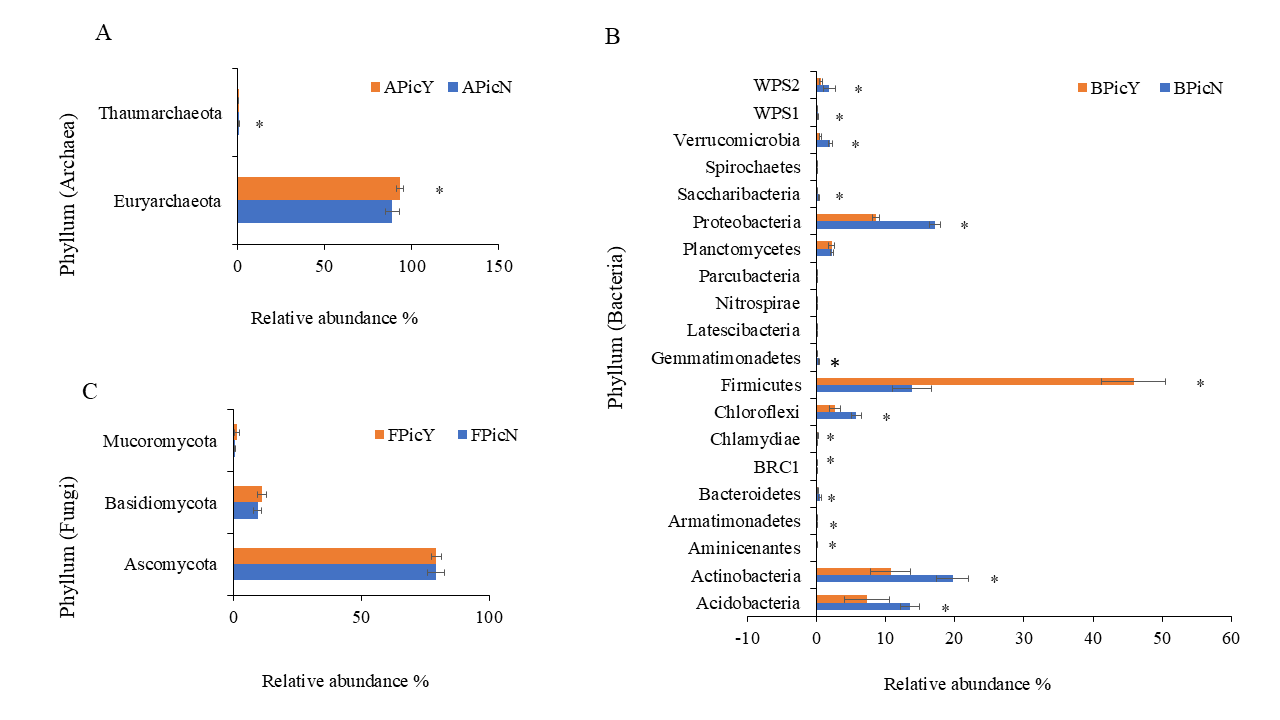

Supplement: S3 Fig — (A) Archaea; (B) Bacteria; (C) Fungi. *FDR adjusted q-value <0.05 (Pesticide—presence (PicY) vs. absence (PicN); Mann-Whitney U test). (TIF) [file pone.0314492.s003.tif]

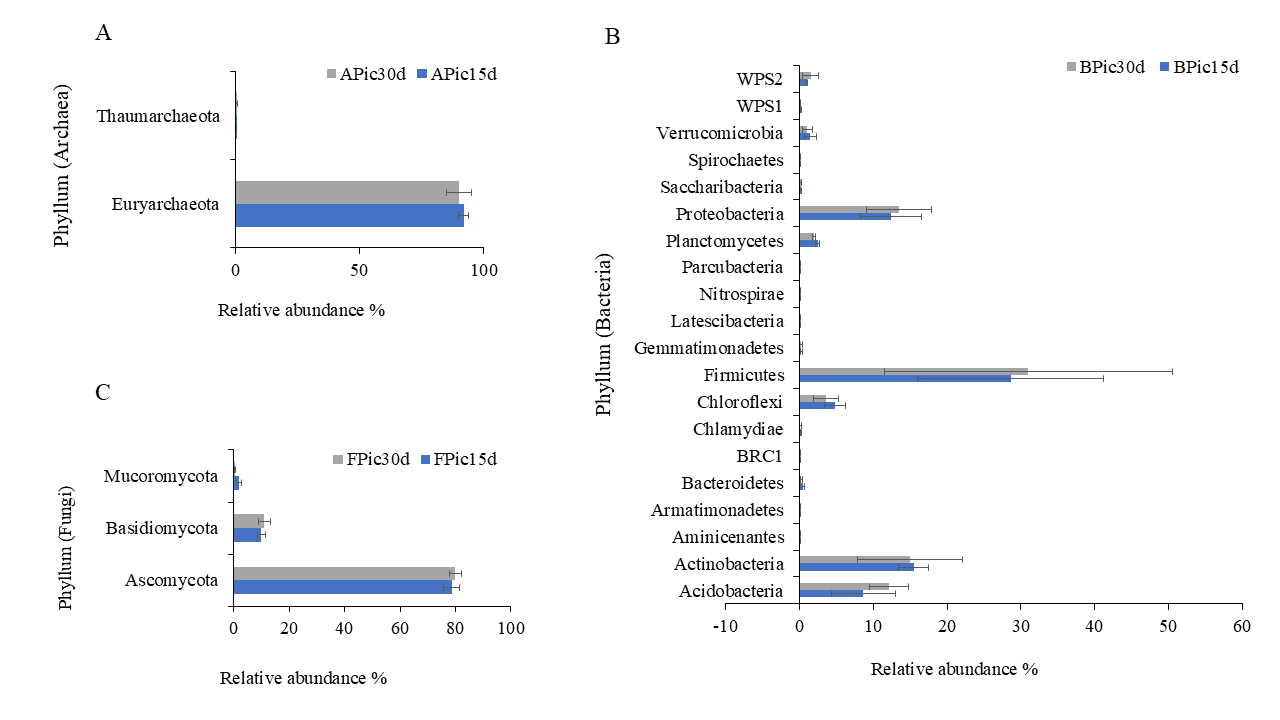

Supplement: S4 Fig — (A) Archaea; (B) Bacteria; (C) Fungi. *FDR adjusted q value <0.05 (Time—15 days (Pic15d) vs. 30 days (Pic30d); Mann-Whitney U test). (TIF) [file pone.0314492.s004.tif]

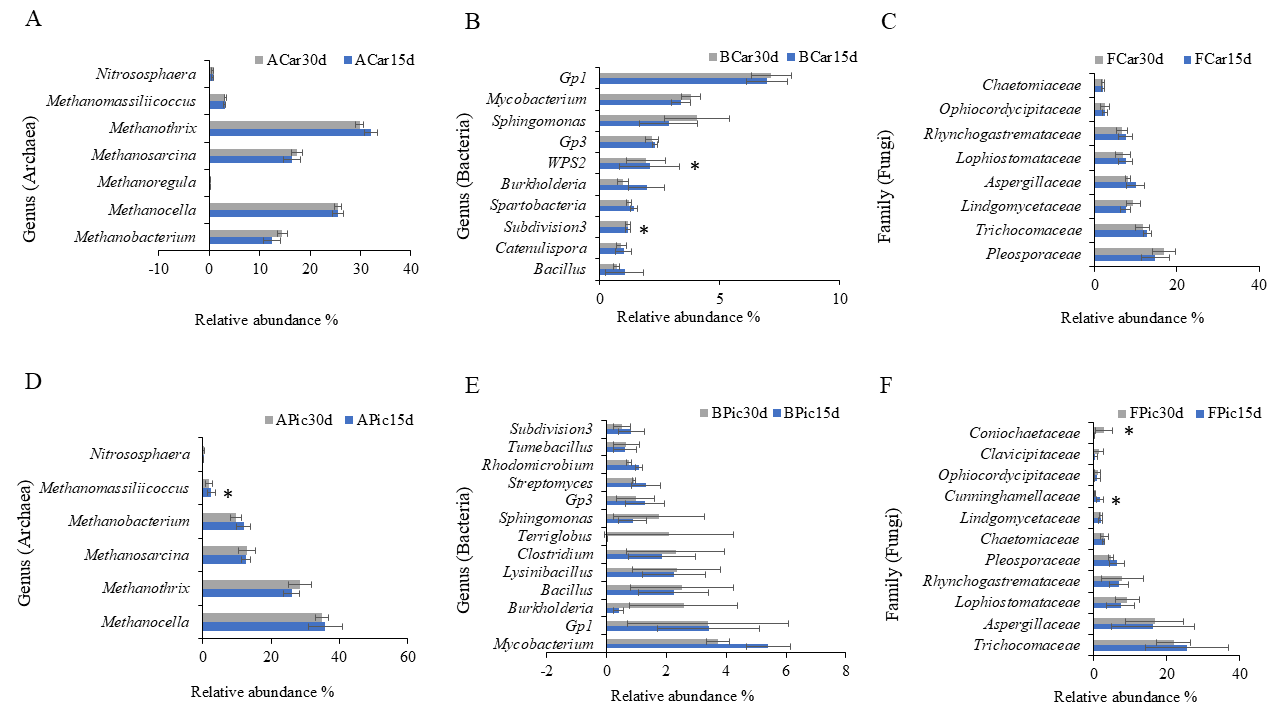

Supplement: S5 Fig — (A and D) Archaea; (B and E) Bacteria; (C and F) Fungi. *FDR adjusted q-value <0.05 (Time—15 days (Car15d; Pic15d) vs. 30 days (Car30d; Pic30d); Mann-Whitney U test). (TIF) [file pone.0314492.s005.tif]

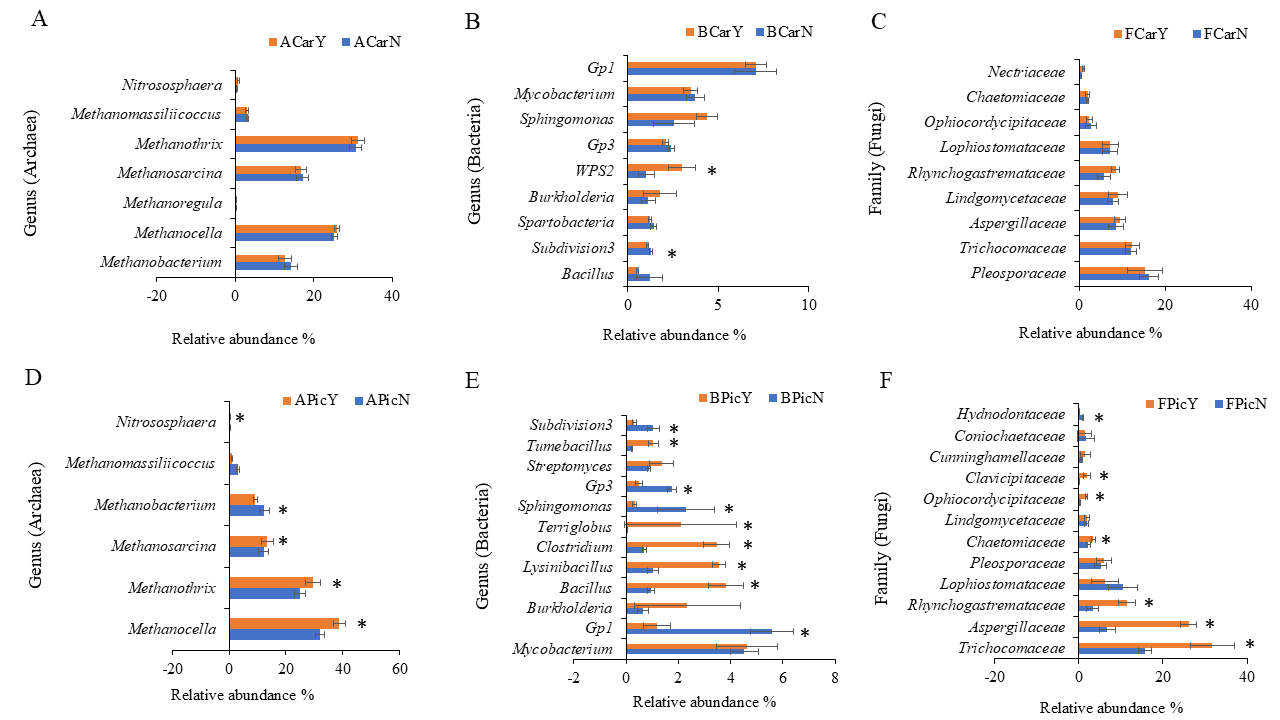

Supplement: S6 Fig — (A and D) Archaea; (B and E) Bacteria; (C and F) Fungi. * FDR adjusted q-value < 0.05 (Pesticide—presence (CarY; PicY) vs. absence (CarN; PicN); Mann-Whitney U test). (TIF) [file pone.0314492.s006.tif]

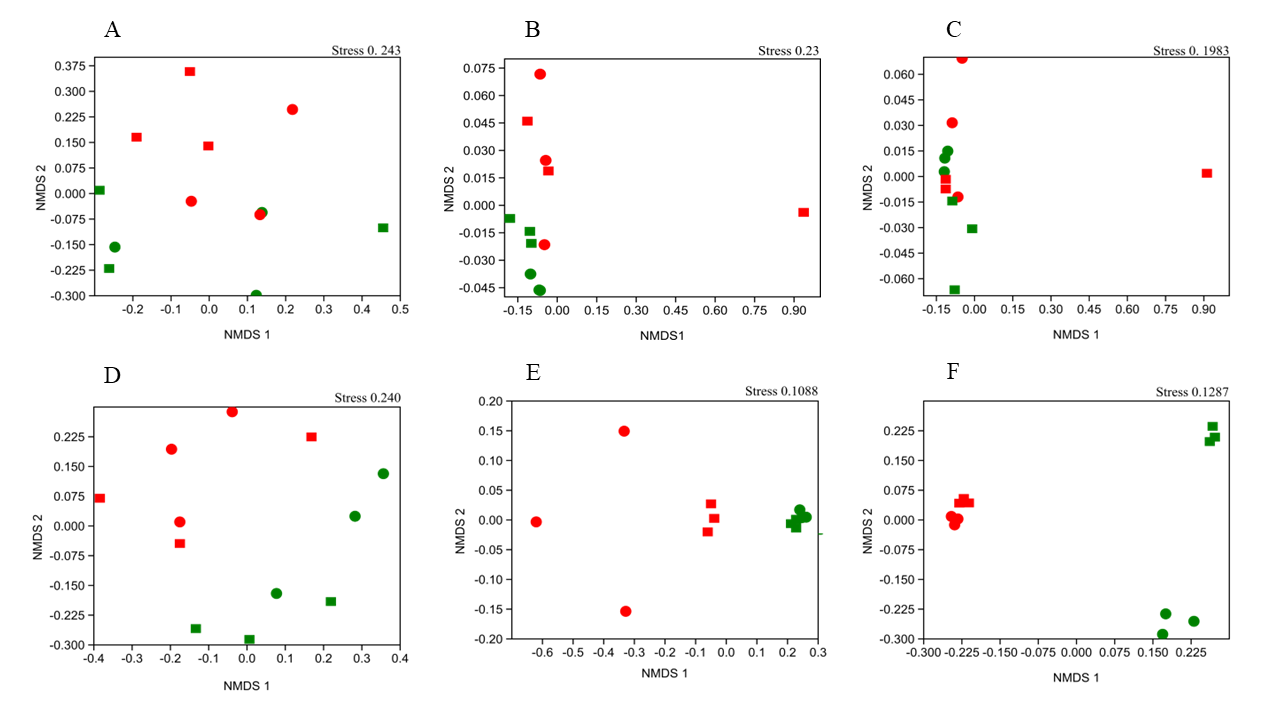

Supplement: S7 Fig — (A and D) Archaea; (B and E) Bacteria; (C and F) Fungi. The pesticide presence and absence are represented in red and green, respectively. The experimental times of 15 and 30 days are represented as rectangles and circles, respectively. (TIF) [file pone.0314492.s007.tif]

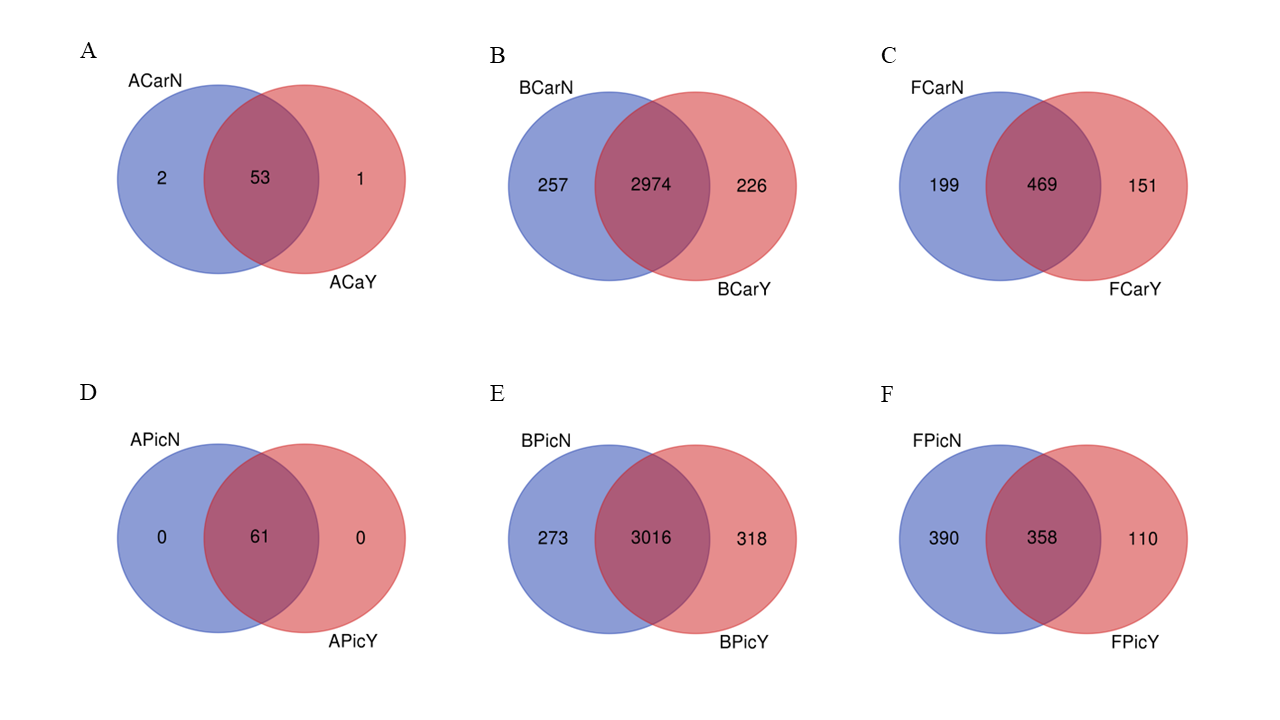

Supplement: S8 Fig — (A and D) Archaea; (B and E) Bacteria; (C and F) Fungi. Analysis of pesticide presence (CarY; PicY) vs. absence (CarN; PicN). (TIF) [file pone.0314492.s008.tif]

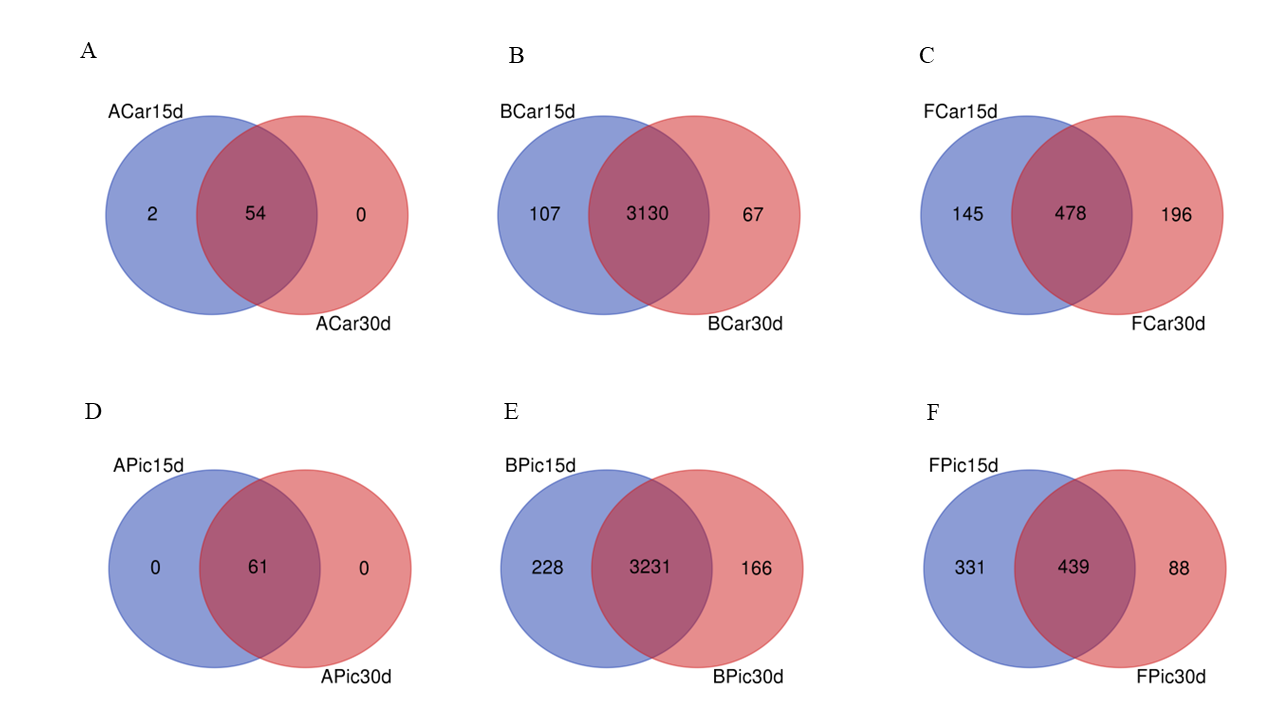

Supplement: S9 Fig — (A and D) Archaea; (B and E) Bacteria; (C and F) Fungi. Analysis of experimental time of 15 days (Car15d; Pic15d) vs. 30d (Car30d; Pic30d). (TIF) [file pone.0314492.s009.tif]

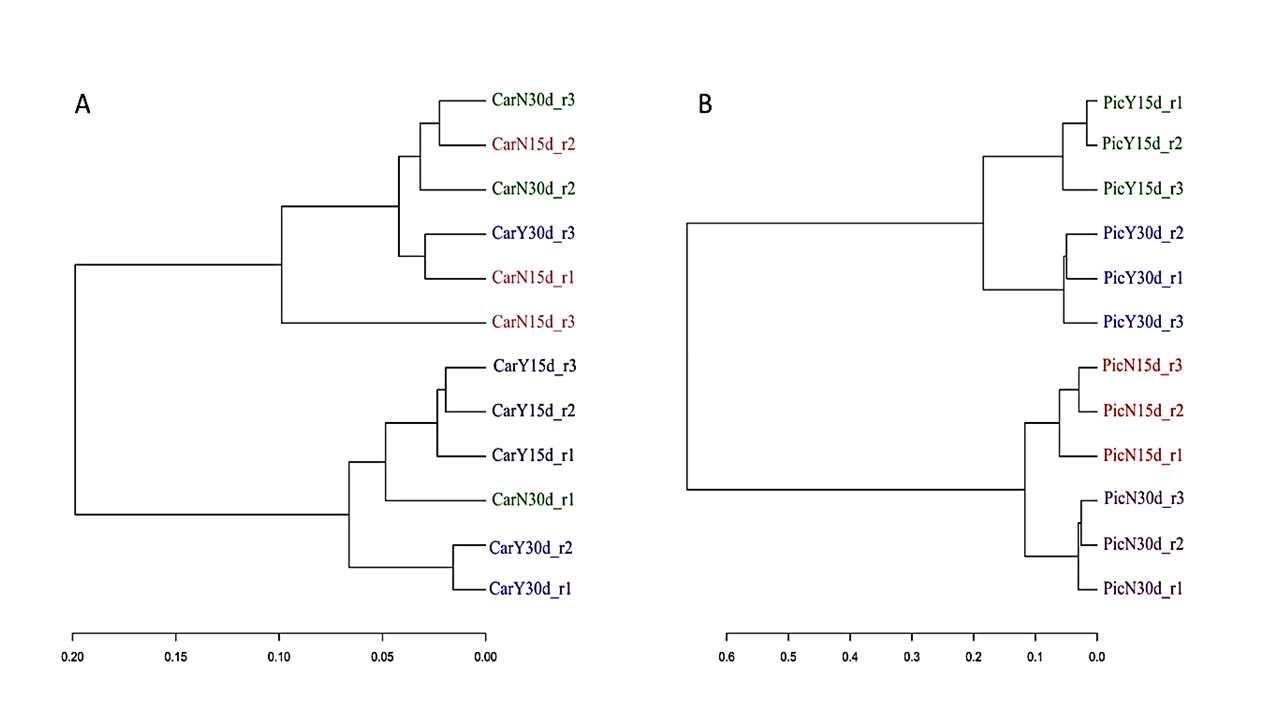

Supplement: S10 Fig — (A) Carbofuran; (B) Picloram. (TIF) [file pone.0314492.s010.tif]

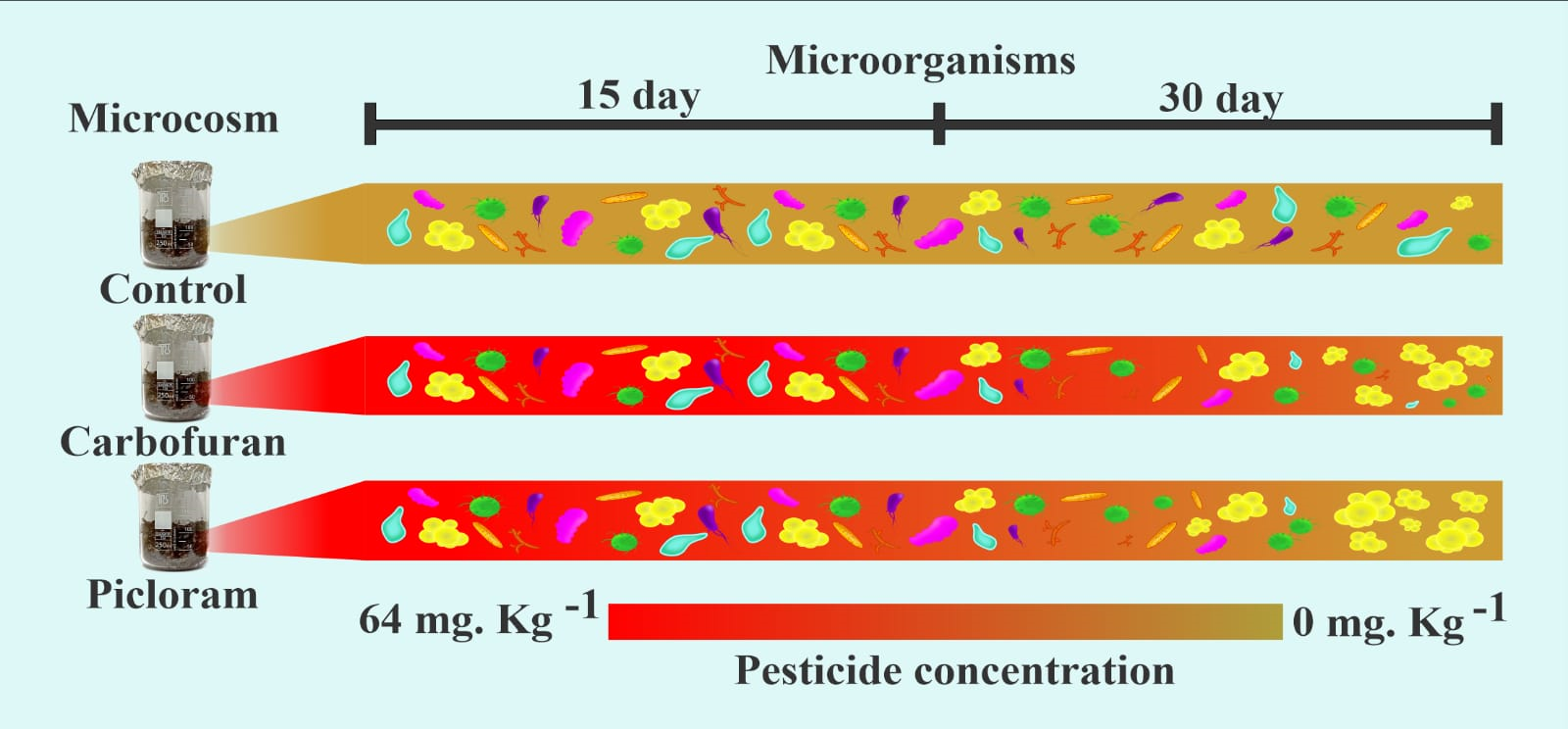

Supplement: S1 Graphical abstract — (TIFF) [file pone.0314492.s012.tiff]
