# Supplementary material for: The pesticides carbofuran and picloram alter the diversity and abundance of soil microbial communities
Source: PLoS One. 2024 Nov 26;19(11):e0314492. doi: 10.1371/journal.pone.0314492 (PMC11594414; doi:10.1371/journal.pone.0314492)
Supplement: Attachment — Submitted filename: Evaluation_Report.docx [file pone.0314492.s013.docx]

**Evaluation**

The research manuscript PONE-D-24-20226 have been submitted to Plos one with title “The pesticides carbofuran and picloram alter the diversity and abundance of soil microbial communities”. The authors investigated the effect of the stress response to the pesticides carbofuran and picloram on soil microbial diversity using DNA metabarcoding. The effect was observed in 24 soil samples, where a total of 118 archaeal, 6,931 bacterial, and 1,673 fungal OTUs were annotated with 97% identity. The study found that the soil diversity and function are affected by the presenceof pesticides and experiment duration. In my opinion, though the manuscript contains well-spelled hypotheses and made use of standard procedures. I however recommend the acceptance of the article with major revisions, as some points need to be addressed before this manuscript is suitable for final publication.

The authors need to:

1. Check the similarity index of the manuscript. The percentage similarity is high to be published in the journal (44% with references, 22% without references). I will suggest the affected part should be reworded.
2. Grammatical presentation should be reviewed throughout the manuscript.
3. The authors did not follow the journal format

Specific comments

i. Abstract contains no methodology
ii. I will suggest the introduction be rewritten in such a way that it will flow logically by consolidating the scattered information about the negative impacts of pesticides which should be merged to form a paragraph.
iii. There are insufficient literatures to back your claim on the role of pesticides on the structure and diversity on the soil microbiota.
iv. At what time/period were the soil samples collected at the region? The depth and diameter the soils were taken should be indicated.
v. Also, the temperature and the cumulative rainfall should also be stated.
