# Supplementary material for: The pesticides carbofuran and picloram alter the diversity and abundance of soil microbial communities
Source: PLoS One. 2024 Nov 26;19(11):e0314492. doi: 10.1371/journal.pone.0314492 (PMC11594414; doi:10.1371/journal.pone.0314492)
Supplement: Attachment — Submitted filename: Answ rev.docx [file pone.0314492.s014.docx]

Oct 04, 2023

Dear Dr. Luigimaria Borruso

**Academic Editor**

**PLOS ONE**

We thank you and the reviewer for dedicating your time to revise our manuscript (PONE-D-24-20226) and make important considerations and corrections, which greatly improved its quality. We corrected the manuscript according to your suggestions and answered to all the points raised. We have sent a marked-up copy of our manuscript that highlights changes made to the original version, and an unmarked version of your revised paper without tracked changes.

We format the manuscript according to PLOS ONE's style requirements.

We insert our collection authorization into the material and methods.

We update and correct the grant information.

All data are available in public databases or described in the manuscript.

The authors agree with the corrected version.

We hope that the revised version fits the standards of Plos One.

Best regards,

Marcos Antônio Soares

Department of Botany and Ecology, Federal University of Mato Grosso

Tel: +55-65-36158870

Email: [drmasoares@gmail.com](mailto:drmasoares@gmail.com)

Reviewer #1

**ANSWER:** We appreciate your dedication in reading the article and providing feedback to the editor. Your suggestions were important for improving the manuscript.

The vertical axis is not found in Figure 1. All figures have this problem.

**ANSWER:** We corrected Figure 1. We insert the vertical axis. Figure 2 has the vertical axis (NMDS2).

Line 98-99: mg kg-1

**ANSWER:** We corrected it: mg kg ^-1^

Line 194-198: Why is there no significance analysis in the pesticide analysis section?

**ANSWER:** We will enter information about the statistical test:

Line 214-269: The results are divided into too many paragraphs and should be consolidated.

**ANSWER:** We consolidate the paragraphs.

Line 259, 264, 266: The format of the "P" value should be uniform, generally uppercase plus italics, with specific reference to journal requirements.

**ANSWER:** We change "p" to "*P*" throughout the text.

Line 256-273: I suggest that results of diversity should be moved before the results of community composition.

**ANSWER:** Dear reviewer, we would like to count on your understanding in maintaining the alpha diversity results after describing the community composition.

Alpha diversity estimators take into account the composition and abundance of species, and we understand that the reader needs to know the composition to then glimpse the alpha diversity indicators. Furthermore, the alpha diversity results are strategically placed before the beta diversity results (analyzed by the Jaccard and Bray-Curtis similarity indices). We believe that the results have more cohesion and are more didactic in that order.

Line 308: The logical structure of the discussion sections needs further combing, and they have too many paragraphs and need to be properly consolidated and reorganized.

**ANSWER:** We consolidate and restructure the paragraphs.

Line: 402-412: The results section needs to be divided into a separate section, and the summary of the results is too shallow and does not highlight the main point. It is well known that the presence of pesticides can change soil microbes, and how to improve them is the focus. Using the metabolome or others?

**ANSWER:** The results are divided into 4 sections to facilitate readers' understanding. We do not use metabolome methods because our hypothesis and objectives are not focused on analyzing microbial community structure. We used the PICRUSt tool to analyze the impact of pesticides on microbiota functionality. This tool allows inference of the functional profile of a microbial community based on marker gene survey along one or more samples..

Line 406-408: This part of the content can be deleted.

**ANSWER:** We have deleted it.

Fig S1: The error bar in Figure S1 B?

**ANSWER:** We have corrected it.

The font and format of all figures need to be uniform.

**ANSWER:** We have corrected it.

I think the full text of the language needs to be further polished by professionals.

**ANSWER:** We send the manuscript to a professional specialized in English language proofreading.

Reviewer #2:

The research manuscript PONE-D-24-20226 have been submitted to Plos one with title “The pesticides carbofuran and picloram alter the diversity and abundance of soil microbial communities”. The authors investigated the effect of the stress response to the pesticides carbofuran and picloram on soil microbial diversity using DNA metabarcoding. The effect was observed in 24 soil samples, where a total of 118 archaeal, 6,931 bacterial, and 1,673 fungal OTUs were annotated with 97% identity. The study found that soil diversity and function are affected by the presence of pesticides and experiment duration. In my opinion, though the manuscript contains well-spelled hypotheses and makes use of standard procedures,. I, however, recommend accepting the article with major revisions, as some points need to be addressed before this manuscript is suitable for final publication.

**ANSWER:** The authors thank you for your dedication in reading and pointing out improvements in the quality of the manuscript.

The authors need to:

Check the similarity index of the manuscript. The percentage similarity is high to be published in the journal (44% with references, 22% without references). I will suggest the affected part should be reworded.

Grammatical presentation should be reviewed throughout the manuscript.

The authors did not follow the journal format

**ANSWER:** We made the necessary adjustments.

We reviewed the text and used two tools to check for plagiarism. The tools are:

https://plagiarismdetector.net/

https://www.plagium.com/

The results indicate that there is no plagiarism in the article.

Specific comments

i. Abstract contains no methodology

**ANSWER:** We insert general information about the methodology.

ii. I will suggest the introduction be rewritten in such a way that it will flow logically by consolidating the scattered information about the negative impacts of pesticides which should be merged to form a paragraph.

**ANSWER:** We rewrote the impact of pesticides on soil microbiota, including other references.

iii. There are insufficient literatures to back your claim on the role of pesticides on the structure and diversity on the soil microbiota.

**ANSWER:** We included other references in the discussion that assessed the impact of other pesticides on soil microbiota. We kept the references already included in the discussion that support the role of pesticides in the structure of the microbial community

iv. At what time/period were the soil samples collected at the region? The depth and diameter the soils were taken should be indicated.

**ANSWER:** We include this information in the material and methods. “Composite samples were obtained from the soil surface (10 cm deep), homogenized, and sieved through a sieve (2 mm mesh).”

v. Also, the temperature and the cumulative rainfall should also be stated.

**ANSWER:** We include this information in the material and methods.
